# Assessment of Fish Diversity in the Ma’an Archipelago Special Protected Area Using Environmental DNA

**DOI:** 10.3390/biology11121832

**Published:** 2022-12-15

**Authors:** Yuqing Wang, Xunmeng Li, Xu Zhao, Jianqu Chen, Zhenhua Wang, Lili Chen, Shouyu Zhang, Kai Wang

**Affiliations:** 1 College of Marine Ecology and Environment, Shanghai Ocean University, Shanghai 201306, China; 2 Engineering Technology Research Center of Marine Ranching, Shanghai Ocean University, Shanghai 201306, China

**Keywords:** biodiversity, environmental DNA, island ecosystem, Ma’an Archipelago, special protected area

## Abstract

**Simple Summary:**

With the development of molecular techniques, environmental DNA (eDNA) methods are increasingly being applied to assess marine fish biodiversity. Fish biodiversity survey methods have been difficult to standardize due to the diversity and complexity of reef habitats. Traditional surveys can damage island ecosystems. A rational method for surveying fish biodiversity in different habitats is urgently needed. This study aimed to investigate the practical validity of the eDNA method for evaluating the fish composition and diversity in different habitats. Additionally, compared with traditional surveys, the eDNA method includes the same results, but also includes species that would not be caught by traditional surveys due to the technical limitations of traditional surveys. This is significant for continuous biodiversity monitoring and management in protected areas.

**Abstract:**

This study aimed to investigate the practical validity of the environmental DNA (eDNA) method for evaluating fish composition and diversity in different habitats. We evaluated the fish composition and diversity characteristics of seven different habitats in the Ma’an Archipelago Special Protected Area in April 2020. The results showed that a total of twenty-seven species of fishes belonging to six orders, eighteen families, and twenty-three genera of the Actinopterygii were detected in the marine waters of the Ma’an Archipelago Special Protected Area. The dominant species in each habitat were *Larimichthys crocea*, *Paralichthys olivaceus*, and *Lateolabrax maculatus*. The mussel culture area had the highest number of species, with 19 fish species, while the offshore bulk load shedding platform had the lowest number of species, with 12 fish species. The rest of the habitat was not significantly different. The results showed that the mussel culture area had the highest diversity index (average value of 2.352 ± 0.161), and the offshore bulk load shedding platform had the lowest diversity index (average value of 1.865 ± 0.127); the rest of the habitat diversity indices did not differ significantly. A comparison with historical surveys showed that the eDNA technique can detect species not collected by traditional methods such as gillnets and trawls. Our study demonstrates the role of eDNA technology in obtaining fish diversity in different habitats and provides a theoretical basis for the continuous monitoring and management of fish biodiversity in protected areas.

## 1. Introduction

Inshore islands and reefs are important components of marine ecosystems, often possessing a variety of habitat types with high primary productivity and abundant bait, and thus providing suitable habitats for many marine organisms [1,2] as well as excellent areas for the development of fisheries and mariculture [3]. Human activities can cause declines in fish diversity or changes in the community structure in these habitats [4]. Reef fish communities are characterized by tropical reef fish groups that can move vertically in depth, depending on the warmth of the ocean. These communities and the resources in island reef waters have been mostly assessed through diving observations [5], gillnetting [6], cage netting [7], electrofishing [8], and small bottom trawling [9]. Most of these traditional sampling methods are limited to economic fish species and specific survey areas, and are costly, time-consuming, and difficult [10]. For various specific life stages and very low-density fish groups, field sampling can be very difficult [11]. The composition and efficiency of catch species vary with different sampling methods, and this affects the accuracy of fish resource assessments on island reefs [7,12]. In addition, offshore cargo-carrying platforms and cage culture areas are prohibited from being surveyed by traditional methods such as trawling and gillnetting [13]. Therefore, choosing a suitable sampling method is key for accurately assessing fish communities in island waters.

Environmental DNA (eDNA) is obtained directly from the DNA of various organisms extracted from environmental samples such as soil, water, glaciers, and sediments [14]. eDNA can reveal past and present-day biodiversity information, and it has been widely used for biodiversity monitoring, including of marine organisms [15,16,17,18]. eDNA provides a method for the comprehensive and systematic monitoring of biodiversity in island reef ecosystems [18]. West et al. [19] used eDNA techniques to analyze fish community compositions in various habitats near southwestern Indian islands and showed that the habitat area-specific variation was correlated with species’ habitat preferences. Giorgio et al. [20] combined three methods, underwater video, fisheries fishing, and eDNA techniques, to investigate fish diversity and found that the eDNA technique was the method that could best reveal species diversity and contained the least redundant fish composition information. This demonstrates that eDNA is an effective tool for monitoring and detecting the diversity of the species in ecosystems. Currently, fish eDNA studies have been widely used in specific ecosystem surveys, and few studies have reported on the use of eDNA techniques for fish diversity surveys in different habitat types. Few researchers have focused on habitats such as rocky reefs and mussel grounds and, to our knowledge, no one has compared the efficiency of eDNA with traditional survey methods for measuring fish diversity in different habitats.

The Ma’an Archipelago Special Protected Area is the core part of the Zhoushan Fishing Ground, which is located in the northeastern part of the Zhoushan Islands in the Zhejiang Province. The Yangtze River water injects into the reserve, bringing many nutrients to the reserve and providing more food for fish [21]. The Taiwan warm current and the coastal cold current converge in the reserve, causing the currents to churn and nutrients to rise [21]. The reserve has many islands and reefs with a variety of habitat types, including rocky reefs, sandy areas, seaweed beds, marine ranching, and a mussel culture area. It is an island-group marine ecosystem dominated by rich marine biological resources and unique natural islands, landforms, reefs, and intertidal wetlands [2], which provide suitable habitat for small coastal estuarine fishes and major economically important fishes [2,22]. To date, most studies have focused on the structure and distribution of fish communities in different habitats in the reserve. Different studies have assessed the characteristics and seasonal variation of fish communities in a variety of habitats in protected areas using a variety of gears, such as trawls and gillnets [23,24]. The results show that different habitats require different nets to obtain fish information, which is not only time consuming, but also dependent on the taxonomic experience of the researchers. We used different nets for surveys in the same habitat, and complete data about the fish were not available [7]. For example, we had difficulties in catching the dominant reef fish *Sebastiscus marmoratus* with trawls and we also had difficulties in catching *Harpadon nehereus* with gillnets [25]. In summary, traditional fishery survey methods not only damage fish habitats, but also require a lot of sampling time and provide incomplete information on fish communities. The eDNA technology and other molecular techniques can be used as new surveying methods for assessing the diversity and community composition of different island reefs, making up for the shortcomings of traditional survey methods.

With the development of massively parallel sequencing technologies, eDNA is being used in a large number of studies for species diversity analyses of monitored areas. Boussarie et al. [26] confirmed that eDNA can be used to detect fish diversity by comparing it with a traditional underwater visual census. In addition, the eDNA quantity was found to be significantly correlated with species abundance or biomass [27]. Stat et al. [28] applied eDNA methods to assess fish diversity in the waters of Coral Bay, northern Australia, and found that the fish species sequences showed a correlation between the number of fish species sequences and the biomass. The choice of both adequate gene markers and related primer pairs affects the eDNA results. The ribosomal small subunit 18S rRNA gene is a common molecular marker for studying eukaryotic diversity in the environment because of its good generality and the abundance of reference sequences in the barcode reference libraries [14]. In this study, we used 18S universal primers and PCR to amplify and sequence eDNA from the Ma’an Archipelago Special Protected Area in order to obtain more accurate biological information. We also used the alpha diversity indices to evaluate the diversity of fish in different habitats. Finally, we compared the similarities and differences between traditional methods and the eDNA technique on the fish information obtained, and we analyzed the advantages and disadvantages of the two methods. This study presents a new method for monitoring and protecting fish resources in protected areas, with a view to provide basic information for the assessment of fish conservation effectiveness and resource management in different habitats.

## 2. Materials and Methods

### 2.1. Sampling Time and Station

**Figure 1:**
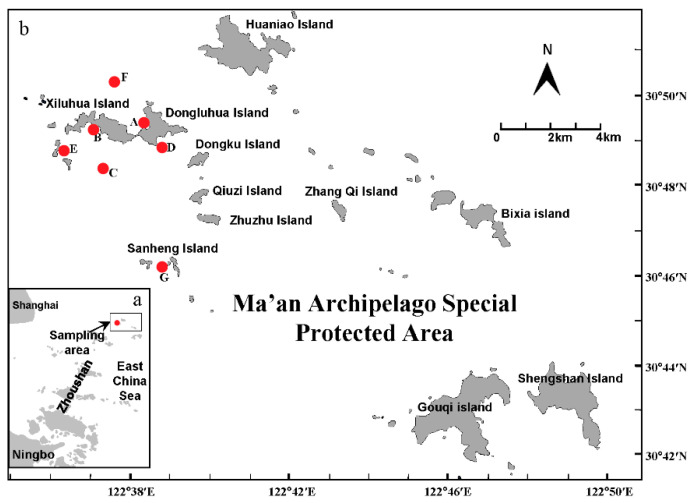
Sampling site map. (**a**) Yangtze estuary and (**b**) Ma’an Archipelago Special Protected Area; the red dots (A~G) are sampling stations.

The survey was conducted in April 2020 (spring) in seven different habitats in the Ma’an Archipelago Special Protected Area (Figure 1). These were the seaweed beds (A), the mussel culture area (B), the offshore bulk load shedding platform (C), the rocky reefs (D), the cage culture area (E), the open sea (F), and the marine ranching area (G).

### 2.2. Water Sample Collection and Processing

At each survey site, three replicates of 1 L of surface water (1 m below the water surface), 1 L of mid-water, and 1 L of bottom water (1 m above the bottom) were collected in sterile sampling bags. The water temperature (T), salinity, pH, and dissolved oxygen (DO) were measured for each layer using a multiparametric probe. The eDNA in seawater was filtered in the laboratory using a mixed-fiber membrane (47 mm diameter, 0.45 μm pore size) immediately after water sample collection. To avoid filter clogging and contaminations, we used pre-filtration with a 1 µm pore size membrane, followed by filtration with a 0.45 µm pore size membrane. Three blank water samples (pure water) were filtered before filtering the experimental water samples to avoid any contamination, and then the experimental water samples were filtered. As we used the mixed-tissue method, water samples from different layers of the same habitat had to be mixed. Therefore, a volume of 3 L of the mixed water samples was filtered from each site by each membrane per replicate, and the filtered membranes were sealed in 1.5 mL sterile centrifuge tubes by curling them with forceps, marked with the sites and sampling times, and stored in liquid nitrogen at −80 °C until DNA extraction. To avoid cross-contamination, the experimental equipment was disinfected and cleaned before and after each filtration.

### 2.3. Environmental DNA Testing

We used the DNeasy Blood & Tissue Kit (Qiagen, Dusseldorf, Germany/cat. 69506) and followed the instructions to extract the DNA from the filter membrane. The extraction process was carried out according to the kit instructions and repeated three times for each sample. The eDNA was quantified using the Qubit 3.0 DNA Assay Kit (Invitrogen, Carlsbad, CA, USA/cat. Q33226), and the quantification method was adjusted according to the kit instructions. The extracted eDNA was mixed and dispensed, and its quality was checked using agarose gel electrophoresis and a NanoDrop2000 spectrophotometer (Thermos Scientific, Waltham, MA, USA).

#### PCR Amplification and Sequencing

We used 18S universal primers (18SF: 5′-GGCAAGTCTGGTGCCAG-3′ and 18SR: 5′-ACGGTATCTRATCRTCTTCG-3′) for the PCR amplification of the environmental DNA samples [29]. The amplification system comprised 35 μL, including 2 × Hieff^®^ Robust PCR Master Mix, 15 μL; PCR products, 10–20 ng; 1 μL each of forward and reverse primer; 2 μL of template DNA; 12 μL of distilled water; and 30 μL of total volume. Paired-end libraries were prepared using a two-step PCR method. PCR thermal cycling was performed after an initial denaturation at 94 °C for 3 min, followed by 35 cycles of (1) denaturation at 94 °C for 30 s, (2) annealing at 45 °C for 20 s, (3) extension at 72 °C for 10 s, followed by one cycle, and finally extension at 72 °C for 5 min, followed by cooling at 10 °C for 5 min. The solution was then kept at 10 °C and transferred to a refrigerator until further use. A negative control was also established to detect microbial contamination from the environment or reagents. Finally, the libraries were double-end sequenced on the Illumina MiSeq platform (commissioned from Shanghai Meiji Biomedical Technology Co., Ltd., Shanghai, China).

### 2.4. Data Analysis

#### 2.4.1. Fish Species Determination

The raw high-throughput sequencing data were quality clipped and spliced using fastp software (https://github.com/OpenGene/fastp/, accessed on 21 October 2022) on the double-ended raw reads data, and clustered by sequences ≥97% using the UPARSE software [30]. The clustering results were called operational taxonomic units (OTUs) [30]. The obtained OTUs were compared with the NCBI database I (https://www.ncbi.nlm.nih.gov/, accessed on 21 October 2022) [31] for sequence alignment. When using 18S rRNA sequences for an environmental DNA macrobarcoding analysis, researchers generally use 96–100% as the species similarity threshold [32,33]. In this study, only marine fish diversity was analyzed, so non-fish species and freshwater fish species were excluded.

#### 2.4.2. Fish Diversity Analysis

The fish orders, families, genera, and species detected by eDNA sequencing were counted using Excel (Microsoft, Redmond, WA, USA). We used the QIIME2 software to count the number of species, the number of OTUs, and the number of sequences at different taxonomic levels and to calculate the relative abundance of the species sequences. To analyze the compositional characteristics of fishes in different habitats, all fishes were classified into warm-temperature species, cold-temperature species, and cold-water species according to their temperature suitability [34]. All of the fish species were classified into sedentary, offshore-migratory, and estuarine-migratory fishes. All of the fish species were classified into perennial, seasonal, and occasional (or rare) species based on the time scale of fish use of the reef habitats. To understand the relationships between dominant species and environmental factors, and the differences in the spatial distribution of fish communities in different habitats, a redundancy analysis and principal coordinate analysis based on the Bray–Curtis distance were performed using the vegan package and the picante package of the R software [35].

In this study, we also used the R programming language (Auckland, New Zealand) to analyze the sequence abundance of the species, and a diversity analysis was calculated to comprehensively evaluate fish diversity among different habitats. We used the Shannon–Wiener index to evaluate the diversity of the fish community distribution: the higher the Shannon value, the higher the community diversity. The Simpson index was often used to quantify the biodiversity of an ecoregion: the greater the Simpson index, the lower the community diversity. The Chao1 index was used to calculate the abundance of fish community distribution. The Pielou index reflected the evenness of the distribution of different species in the community.

## 3. Analysis of Results

### 3.1. Results of eDNA Sequencing

A total of 21 water samples from seven different habitats in the Ma’an Archipelago Special Protected Area were analyzed, and a total of 697,534 raw sequences were obtained by processing the raw double-ended sequencing data, including 516,785 high-quality sequences with an average length of 265 bp. A total of 1076 OTUs were obtained by clustering at ≥97% sequence similarity. The high-throughput sequencing statistics of the eDNA samples are shown in Table 1.

### 3.2. Fish Species Composition

**Figure 2:**
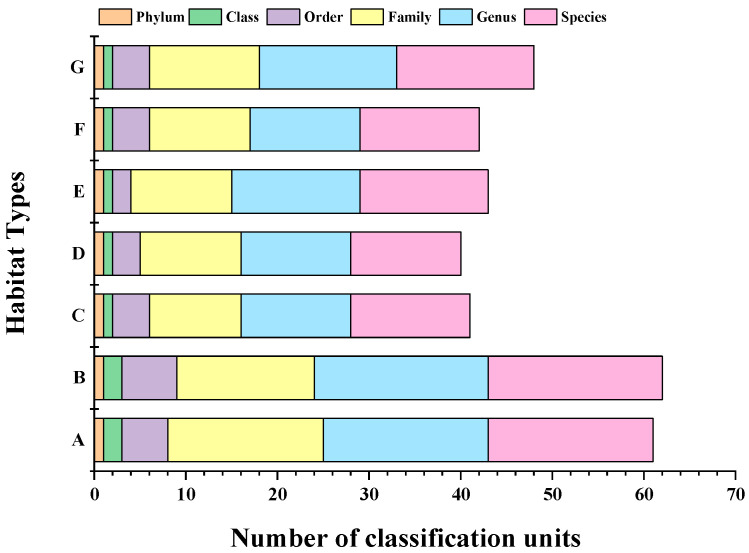
Results of OTU division and taxonomic classification identification of fish.

The OTUs were compared with databases (NCBI); the OTUs of annotated sequences were classified and their taxonomic statuses were identified. A total of twenty-seven species of marine fishes in twenty-three genera of eighteen families and six orders were detected, among which Perciformes was the most abundant with nineteen species (accounting for 70.37% of the detected species), followed by Cypriniformes with three species (accounting for 11.11% of the detected species; Figure 2).

#### 3.2.1. Fish Species Composition in Different Habitats

**Figure 3:**
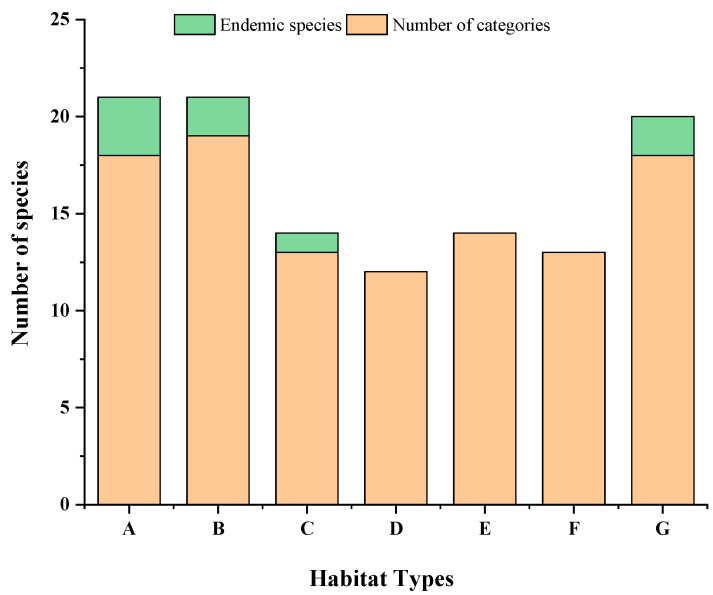
Results of fish OTU delimitation and taxonomic status identification.

There were differences in the fish compositions between different habitats (Table 2, Figure 3). The mussel culture area displayed the highest number of fish species, with 19 species. The seaweed beds and marine ranching were tested for 18 species of fish. The numbers of species in the offshore bulk load shedding platform, the rock reef, the cage culture area, and the open sea area were similar, with 13, 12, 14, and 13 species, respectively.

The endemic species of the seaweed beds were *Scarus ghobban*, *Zoarces viviparus*, and *Scomberomorus niphonius*; the endemic species of the mussel culture area were *Ilisha elongate* and *Nibea albiflora*. *Coilia nasus* was an endemic species of the offshore bulk load shedding platform; *Psenopsis anomala* and *Siganus fuscescens* were endemic to the marine ranching, and there were no endemic species in the rock reef, cage culture area, or open sea.

For all fish species (Table 2), five types of thermochromic fish species were detected, including two species of temperate-water fishes, three species of warm-water fishes, thirteen species of warm-temperature fishes, three species of cold-temperature fishes, and five species of wide-temperature and wide-salinity fishes. We found a total of eleven offshore migratory fish species, one estuarine migratory fish species, and fourteen sedentary fish species. We classified thirteen perennial fish species, ten seasonal fish species, and three occasional fish species based on changes in fish time scales.

#### 3.2.2. Species Diversity Indices of Different Habitats

The mean Simpson index was 0.834 (range: 0.757–0.868), the habitat index values were highest for B and lowest for C, and three habitat index values were above the mean. The mean Shannon–Wiener index was 2.12 (range: 1.865–2.352). The Shannon index was highest in habitat B and lowest in habitat C, with significant differences between habitats. The high-throughput sequencing of all samples showed that the Chao1 index ranged from 74,144 to 96,912, with a maximum B-habitat index of 95.912 and a minimum C-habitat index of 74,141. The Pielou evenness index varied between habitats, with the highest index values in habitat G (average values of 0.829 ± 0.038) and the lowest in habitat C (average values of 0.727 ± 0.033).

#### 3.2.3. Composition of Dominant Fish Species

**Figure 4:**
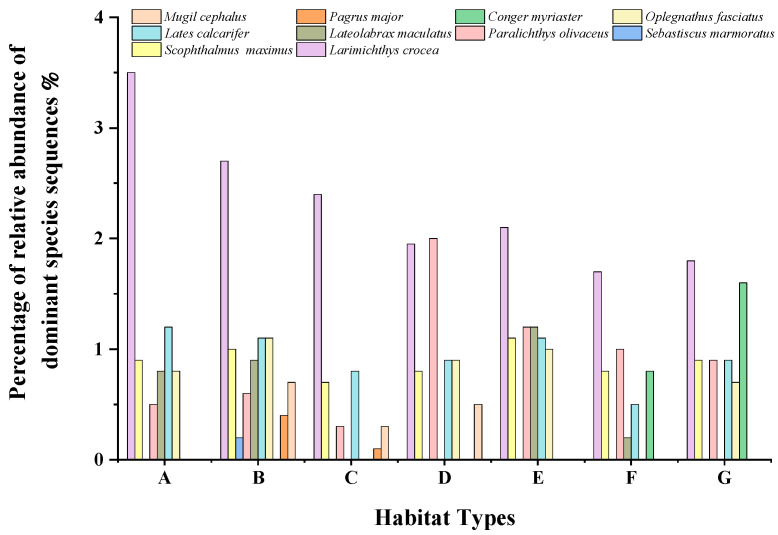
Composition of dominant species at each site.

The top 10 dominant fish species in terms of abundance detected by eDNA analysis were *Larimichthys crocea*, *Lates calcarifer*, *Oplegnathus fasciatus*, *Lateolabrax maculatus*, *Pagrus major*, *Mugil cephalus*, *Scophthalmus maximus*, *Paralichthys olivus*, *Conger myriaster*, and *Sebastiscus marmoratus* (Figure 4).

### 3.3. Correlation Analysis for Each Site

**Figure 5:**
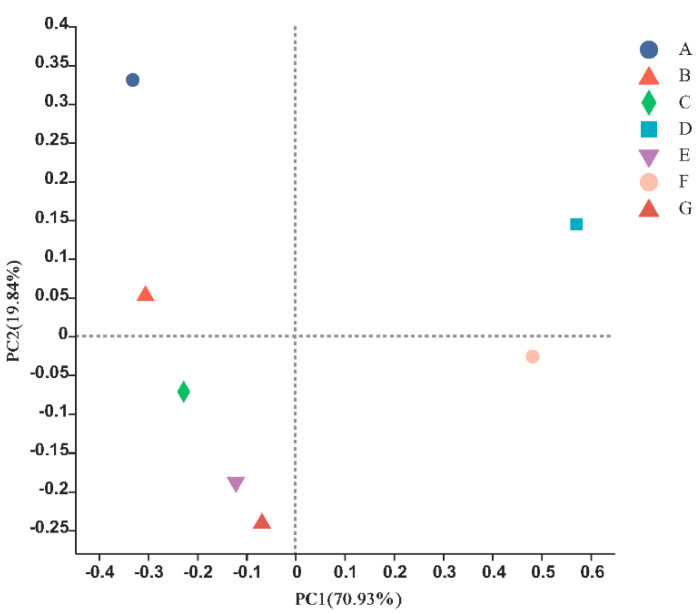
Similarity of fish composition among sites.

The PCoA results based on the Bray–Curtis dissimilarity showed that the fish communities of seven typical habitats in the Ma’an Archipelago Special Protected Area differed and could be divided into five different communities, among which E (cage culture area), C (offshore bulk load shedding platform), and G (marine ranching) were close together and had similar fish structure compositions, and thus could be viewed as one community (Figure 5). The remaining four sites, A (seaweed beds), B (mussel culture area), D (rock reef), and F (open sea), were far away from each other and formed different fish community structures.

**Figure 6:**
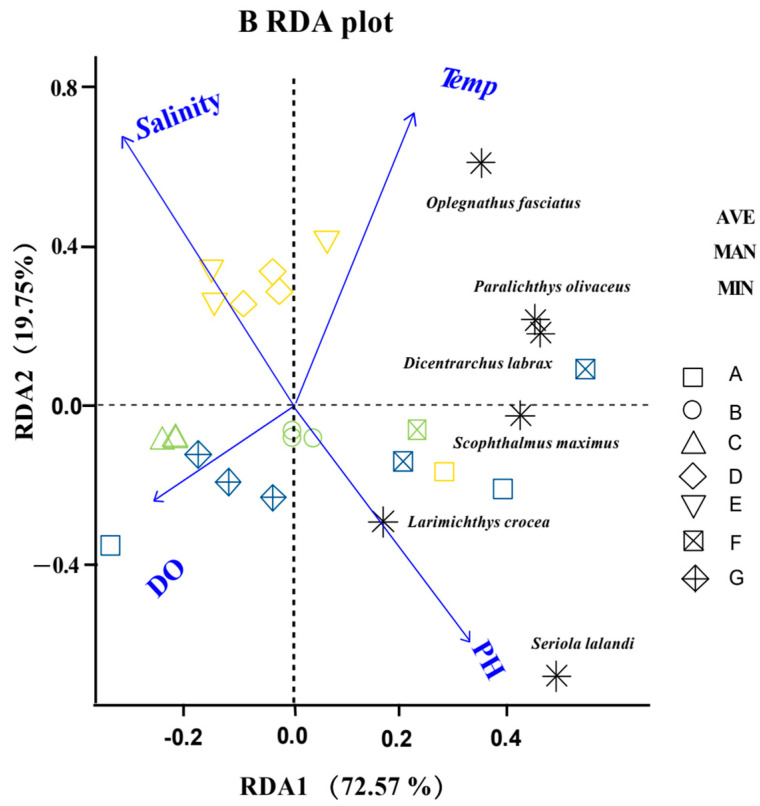
Redundancy analysis of fish species content and environmental factors.

The RDA analysis using environmental factors (T, salinity, DO, and pH) and the sequence richness of dominant species showed that the eigenvalues of the first ordination axis (RDA1) explained 72.57% of the variance (Figure 6). The eigenvalues of the second ordination axis (RDA2) explained 19.75% of the variance (Figure 6). The correlation coefficients of pH and DO with the first ordination axis were 0.51 and 0.24, respectively, which means that along the first axis of the RDA, there were gradual increases in the pH and DO from left to right, and among all four environmental factors, the correlation between pH and the first axis was the largest. The pH and DO were the main influencing factors for the distribution of dominant fish species and fish community structures in the Ma’an Archipelago Special Protected Area when surveyed using eDNA technology. The pH and DO were also the main influencing factors of the fish community structures in A (seaweed beds) and G (marine ranching), and pH was the main influencing factor of the fish community structures in B (mussel culture area) and F (open sea). Salinity was the main influencing factor of the fish community structures in C (offshore bulk cargo shedding platform) and E (cage culture area), and salinity and T were the main influencing factors of the fish community structure in D (rocky reef).

## 4. Discussion

### 4.1. Category Composition

The Ma’an Archipelago Special Protected Area has a wide variety of economic fish species [2], and due to severe environmental damage in recent years, the fishery resources have declined [36]. The basis and prerequisite for research and conservation, and even for the sustainable development and use of these resources, is a clear understanding of the composition and structure of the fish communities in the reserve. The current research methods are destructive to fish habitats and the community composition, are time-consuming and laborious to conduct, and are dependent on the taxonomic basis of the researcher [10,23]. Therefore, we attempted to analyze the fish composition of typical habitats in protected areas using eDNA techniques, and we expect that these methods/results will be useful for subsequent research and conservation measures. A number of economically important fish inhabit the area. Therefore, in this study, we analyzed the fish composition of seven typical habitats in the protected area using eDNA technology. The analysis detected twenty-seven species of fish belonging to twenty-three genera in six orders and eighteen families. The relative sequence abundances of *L. crocea*, *L. maculatus*, and *O. fasciatus* were higher, followed by *S. maximus* and *P. olivaceus*.

The eDNA technique is highly sensitive, not only for the identification of fish diversity in the water column, but also for testing the morphological identification results of some fish species, as well as for the detection of species with very narrow distributions and exotic species [37]. In this study, the eDNA technique was used to identify fishes that have not been reported or were less reported in the Ma’an Archipelago Special Protected Area, such as *S. ghobban*, *L. calcarifer*, and *S. lalandi*. *S. ghobban*, especially the juveniles, mainly inhabit algal thickets [38] and has been found in dive surveys of algal fields in the study area [39]. As a result, the eDNA technique, as a non-destructive survey method, is more labor-saving than traditional methods. It can greatly simplify fish diversity surveys and the monitoring of alien species in marine protected areas.

#### 4.1.1. Comparison of Fish Diversity

The Shannon–Wiener and Simpson indices can indicate the level of fish diversity (Table 3). The Simpson index in this survey was 0.75–0.86, the Shannon index was 1.86–2.35, and their distribution trends were basically the same. Exceptionally, the OTU abundance of fish species can be expressed as the Chao1 index. The results show large differences between individual habitats. These may be due to the influence of individual habitats (C) on the whole, but may also be related to the quality of eDNA and differences in the sampling sites of marine samples. This may be related to the habitat characteristics, as B (mussel culture area) was located in the open sea [40] and contained components such as floating ropes, cultured mussel strings, and floating balls [39], which could have attracted a variety of fish. The C habitat (offshore bulk cargo shedding platform) had lower temperatures than the other habitats due to a lack of light all year round; the diversity of fish species was the lowest in this habitat. When compared to historical survey data, we found that the diversity of fish species obtained by eDNA testing was generally higher than that obtained by traditional methods. This could be seen by comparing the diversity index values of the same survey sites. It can also be shown that eDNA technology can be applied in fish diversity surveys.

#### 4.1.2. Analysis of Fish Species Composition by Different Survey Methods

Lin et al. [41] studied fish diversity in the rocky reef waters of Dachen Island, Taizhou, Zhejiang Province, through eDNA and multi-mesh gillnets. They found that the number of species detected by the eDNA technique was greater than or equal to the number of species captured by multi-mesh gillnets, and the overall effect of detecting species diversity was significantly higher than that of multi-mesh gillnets. In this survey, eDNA technology was used to detect twenty-seven species of fish in twenty-three genera of six orders and eighteen families in seven different island reef habitats, and the number of fish species detected by eDNA technology in marine rangeland habitats was greater than the number of fish species captured by traditional methods (Table 4). The difference may have been due to the following three reasons:

(1) During the long sampling interval, the dominant fish species and community composition in the reserve may have changed. For example, Han et al. [25] investigated the fish community pattern of the reserve using bottom trawling, and found that *Harpadon nehereus*, *Psenopsis anomala*, *Collichthys niveatus*, and *Cynoglossus robutus* were the dominant species in the reserve in spring; compared with the survey by Wang [21], the dominant species had changed, and *Thryssa kammalensis* did not appear among the dominant species in the area. This study showed a change in the dominant fish species in the reserve. *L. crocea* had become the new dominant species, and species such as *H. nehereus* did not appear in this study.

(2) Changes in fish habits and environmental factors may have affected the fish diversity, as this survey was conducted in April, while historical surveys have been conducted in May [21,25,42]. Offshore migratory fish (e.g., *C. niveatus* and *Collichthys lucidus*) will enter the reefs to spawn in the spring and summer seasons when the temperature is higher [25]. The fish diversity of the reserve cannot be well described by a single sampling. Zamani et al. [43] used the eDNA technique to analyze the different fish communities and diversity of the reef and found significant differences in the fish diversity in both summer and winter. Subsequent sampling between different seasons should be carried out for a comprehensive evaluation of fish diversity and community structure in the reserve.

(3) The primer selection in the eDNA assays could have led to differences in comparative databases; for example, some freshwater fish species were detected in this study. The choice of primers also differed from that of other studies. The results of the different primers should be compared when the corresponding survey is carried out later. A suitable primer should be selected for the detection of fish species in the reserve.

### 4.2. Biomass Analysis

Studies have shown that the eDNA abundance of organisms in aquatic ecosystems is well correlated with biomass, and thus is widely used in biomass assessments [8,17,44,45]. In this study, the eDNA analysis showed that the dominant species in spring in the Ma’an Archipelago Special Protected Area were *L. crocea*, *L. calcarifer*, *L. maculatus*, *P. major*, *M. cephalus*, *P. olivaceus*, *C. myriaster*, *S. marmoratus*, and *S. maximus*. These results differ from the findings of Wang [21,23] through gillnets and trawls. For example, *M. miiuy*, *N. albiflora*, and *S. marmoratus*, which had a higher biomass in spring 2009 (April), were less dominant in the present results, and fish such as *T. kammalensis* and *Setipinna tenuifilis* did not appear in this study. The abundances of the dominant species in this survey, *P. olivaceus* and *S. maximus*, were low in the traditional fishery resource survey. These differences may be related to the habits of the target species and the degradation rate of eDNA in the environment. For example, *M. cephalus* is a warm-temperate demersal fish with the habit of sinking during the day and surfacing at night [46]. These patterns may affect the environmental DNA content of otoliths in the samples collected during the day. Therefore, the eDNA survey should fully consider the ecological habits of fishes that may exist in the surveyed sea area and employ an optimized design in terms of the water depth and time of sampling.

The Ma’an Archipelago, located in the center of Zhoushan Fishery, Zhejiang Province, has traditionally been the spring spawning ground of the Daiku Nation of the East China Sea, and the survey period coincided with the spring breeding period of *L. crocea* [47,48]. As a species with declining coastal resources in China, Zhejiang and other provinces and cities have carried out larger-scale stocking activities in recent years to restore *L. crocea* and have achieved certain results [36]. Wang et al. [49] evaluated the fishery resources of *L. crocea* in the East China Sea based on eDNA technology and found that its distribution area and water layer were consistent with the results of traditional trawling. The study demonstrated the feasibility of the eDNA method for surveying and monitoring the natural resources of the greater amberjack (an economically important fish species). In this study, the eDNA test also showed that *L. crocea* was detected in all the surveyed habitats and was the dominant species in the surveyed area, further indicating that the *L. crocea* resources in the Ma’an Archipelago area had recovered well. *P. olivaceus* is an artificially cultured species in the offshore area, and it has a high biomass in the Ma’an Archipelago Special Protected Area [22,50]. Sato [51] assessed the biomass of fish in an artificial reef and the open sea by the eDNA technique, and found that *O. fasciatus* had a large biomass and was the dominant species in the study area, and this study also showed that *O. fasciatus* had a large biomass and was the dominant species in the Ma’an Archipelago Special Protected Area. The biomass of the other dominant species also varies from site to site, which is somewhat related to their habits. Overall, there was some increase in the fish biomass in all habitats.

### 4.3. Differences between Different Habitats

There are many islands and reefs in the Ma’an Archipelago Special Protected Area, with a variety of habitats such as rocky reefs, seaweed beds, and mussel raft culture areas, forming a marine environment with extremely complex spatial and temporal changes in environmental factors such as temperature and salinity [21]. The behavior and distribution of fish are influenced by environmental factors such as the water temperature, water depth, salinity, and dissolved oxygen [52,53]. Studies have shown that the seaweed beds have a buffering effect on the distribution of and changes in the current, pH, dissolved oxygen, and water temperature, and the internal macroalgae and their epiphytes can be used as bait for a variety of marine organisms such as fish, providing them with an excellent habitat [54,55]. The eDNA results showed that a total of 18 fish species were detected in the seaweed beds, including *L. crocea*, *A. schlegelii*, and *O. fasciatus*. The fish community structure was influenced by two environmental factors, pH and DO, and the respiration of the algal species supported by the seaweed farm during the survey period may have affected the changes in pH and DO [55,56]. This change occurred accordingly in our marine environmental surveys. The rocky reef habitat is a “natural harbor” for fish community reproduction, development, feeding and protection from enemies, and is dominated by fish that prefer benthic animals and algae [6,57], such as *O. fasciatus* and *A. schlegelii*. The eDNA results showed that *O. fasciatus* and *A. schlegelii* were the dominant species in the rocky reef habitat, while changes in salinity and temperature affected the community structure composition of this habitat. This result is also related to a study by Wang [48] on the environmental factors affecting the changes in fish community patterns and compositions in the Ma’an Archipelago Special Protected Area.

Studies have shown that rocky reef waters with an abundant seaweed attachment have higher fish diversity than muddy or sandy habitats without seaweed [58], and the density of fish resources is much higher in near-reef areas than in far-reef areas [55,59]. In this study, the fish diversity in the open sea and the cage culture habitats were lower than that in algal fields. Marine bulk reduction platforms, mariculture nets, and marine buoys are important infrastructure elements for marine economic development and resource exploitation [60], and a large number of fouling organisms such as barnacles and mussels are often attached to the structures [59]. In this study, we found that *A. schlegelii* had a larger biomass in the platform, while the stomach content analysis also showed that there were more barnacles and mussels in the stomach, and thus the offshore bulk load shedding platform provided a good habitat for *A. schlegelii*.

Artificial reefs are one or more natural or artificial structures placed on the seabed [61]. As an important initiative for the restoration of offshore biological resources, they combine the characteristics of natural and artificial environments, and their unique fish aggregation patterns have complementary and reinforcing effects on the natural community structure, playing an active and special role in the conservation of rocky reef resources and island biodiversity [24,62]. Some studies have found that artificial reefs are higher than natural rock reefs and offshore platforms in terms of species richness, population density, and diversity [63,64,65]. This study also found that the number of fish species, the diversity, and the biomass in the marine ranching were higher than in rock reefs and the bulk load shedding platform, indicating that artificial reefs in the marine ranching play the role of fish attraction and the effect of ecological protection. Wang et al. [21] found that temperature was the largest correlate of fish communities in the marine ranching area. This study showed that the pH and DO of water bodies were the main influencing factors of the fish community structure in the marine ranching area, probably due to the fact that this study employed only a single sampling, without regard to seasonal variation, and the temperature variation was low.

In summary, the differences between different areas resulted in the formation of reef ecosystems [6]. Biological surveys are easily hindered, and fish surveys based on traditional methods often require multiple voyages, multiple combinations of nets, and factors that consume large amounts of human and material resources [10]. They are also constrained by the weather, fish habits, and the professional abilities of researchers (e.g., diving, taxonomy) [14]. The eDNA technique largely avoids these limitations, reduces the biological damage caused by the survey, increases flexibility and sensitivity, and can complement traditional methods for biological surveys of island ecosystems [17,18,45].

## 5. Conclusions

Accurate information on the composition, distribution, and abundance of major fish communities is needed for fisheries management and resource conservation. We found that the fish community compositions and structures differed among ecological types, and that eDNA concentrations of the same species differed significantly among habitat types, indicating a significant horizontal distribution of fish. We also determined the effect of environmental factors such as SST and salinity on fish distribution, which has predictive value for fish distribution. This study verifies the feasibility of eDNA. In the future, eDNA technology will be used in the Ma’an Archipelago Special Protected Area to conduct fishery resource surveys for different island habitat types, to provide information support for fish diversity conservation in the protected area, and to provide a scientific basis for carrying out fish resource management and detection in the protected area. At the same time, the influence of environmental complexity on the detection results of eDNA technology should be fully considered, and the influence of the environment on it should be reduced by improving the precision and increasing the sampling time.

## Figures and Tables

**Table 1:** Sequence quantity (eDNA) results from each station for fish species composition.

Habitat Type	Sample Label	Total Number of Bases Sequenced	Accounted for	Effective Sequence
A	A1	4,457,369	94.35	52,440
	A2	4,563,778	93.97	53,692
	A3	5,254,263	94.15	61,815
B	B1	5,784,349	94.40	68,051
	B2	4,042,183	94.74	47,555
	B3	4,118,932	93.73	48,458
C	C1	4,325,293	95.41	50,886
	C2	4,301,465	94.62	50,605
	C3	3,877,771	93.90	45,621
D	D1	4,330,038	93.58	50,942
	D2	4,178,398	94.54	49,158
	D3	4,064,025	93.18	47,812
E	E1	3,753,436	93.70	44,158
	E2	3,845,085	95.75	45,236
	E3	4,239,784	93.25	49,880
F	F1	5,016,051	94.58	59,012
	F2	4,285,021	94.87	50,412
	F3	4,031,488	94.86	47,429
G	G1	5,361,963	94.83	63,082
	G2	4,028,498	94.05	47,394
	G3	4,428,378	94.31	52,099

**Table 2:** Fish species and ecotypes detected by environmental DNA.

Order and Species	Habitat Type							Ecological Types
	A	B	C	D	E	F	G	
Anguilliformes								
1 *Conger myriaster*		+		+		+	+	**▲●
Clupeiformes								
2 *Engraulis japonicus*	+	+	+			+		**▲●
3 *Coilia nasus*			+					*△●
4 *Ilisha elongata*		+						***▲●
Perciformes								
5 *Johnius belangerii*					+	+	+	*◆○
6 *Larimichthys polyactis*			+			+	+	*▲●
7 *Larimichthys crocea*	+	+	+	+	+	+	+	*▲●
8 *Nibea albiflora*		+						*▲●
9 *Miichthys miiuy*	+	+		+	+		+	*▲●
10 *Pagrus major*		+	+		+		+	***◆○
11 *Acanthopagrus schlegelii*	+	+	+		+		+	□◆○
12 *Oplegnathus fasciatus*	+	+	+	+	+	+	+	*◆○
13 *Scomberomorus niphonius*	+							*◆○
14 *Psenopsis anomala*							+	*◆○
15 *Zoarces viviparus*	+							****◆○
16 *Lateolabrax maculatus*	+	+		+	+	+		□▲●
17 *Mugil cephalus*	+	+		+	+		+	□◆○
18 *Lates calcarifer*	+	+	+	+	+	+	+	*▲◎
19 *Dicentrarchus labrax*	+	+	+	+	+	+	+	◎
20 *Sparus aurata*	+	+	+	+	+	+	+	□◆◎
21 *Seriola lalandi*	+	+	+	+	+	+	+	*▲●
22 *Scarus ghobban*	+							◎
23 *Siganus fuscescens*							+	□◆○
Scorpaeniformes								
24 *Sebastiscus marmoratus*	+	+					+	*◆○
Pleuronectiformes								
25 *Paralichthys olivaceus*	+	+	+	+	+	+	+	***◆○
26 *Scophthalmus maximus*	+	+	+	+	+	+	+	*****◆○
Tetraodontiformes								
27 *Takifugu niphobles*	+	+						*◆○
Number of categories	18	19	13	12	14	13	18	
Special Species	3	2	1	0	0	0	2	

Note: +: occurrence; * warm-temperature species; ** warm-water species; *** warm water; **** cold-temperature species; ***** cold-water species; ▲ offshore migration; △ estuarine migration; ◆ sedentary; ◎ occasional species; ● seasonal; ○ perennial species; □ wide temperature and wide salinity.

**Table 3:** Alpha diversity indices of relative abundances of fish species.

Habitat Type	Alpha Diversity Indices			
	Shannon–Wiener Diversity Index	Simpson Diversity Index	Chao1 Index	Pielou Evenness Index
A	2.274 ± 0.163	0.839 ± 0.034	86.671 ± 7.178	0.786 ± 0.029
B	2.352 ± 0.161	0.868 ± 0.037	95.912 ± 7.581	0.799 ± 0.031
C	1.865 ± 0.127	0.757 ± 0.036	74.141 ± 4.997	0.727 ± 0.033
D	1.993 ± 0.102	0.826 ± 0.018	76.544 ± 4.657	0.802 ± 0.017
E	2.106 ± 0.100	0.846 ± 0.019	81.781 ± 4.462	0.798 ± 0.020
F	2.000 ± 0.122	0.816 ± 0.023	77.505 ± 5.425	0.779 ± 0.024
G	2.245 ± 0.113	0.863 ± 0.013	88.352 ± 4.415	0.829 ± 0.038

**Table 4:** Number of fish species in different survey methods.

Habitat Type	eDNA *	Bottom Trawling [21]		Bottom Trawling (Unpublished Data)		Bottom Trawling [42]		Multiple Gillnet [22]	
	Species *	Species	Specific Name	Species	Specific Name	Species	Specific Name	Species	Specific Name
Oceanic ranch	18	9	*N. lbiflora* *L. polyacti* *S. marmoratus* *J. belangerii* *Hexagrammos indicus* *Chelidonichthys kumu* *P. olivaceus* *T. kammalensis* *C. nasus*	7	*Engraulis japonicus* *J. belangerii* *M. miiuy* *P. anomala* *Pampus argenteus* *L. polyacti* *C. lucidu*	5	*J. belangerii* *L. polyacti* *S. marmoratus* *Stephanolepis cirrhifer* *P. olivaceus*	4	*S. marmoratus* *T. kammalensis* *M. miiuy* *A. schlegelii*
Seaweed beds	18	6	*N. albiflora* *S. marmoratus* *L. maculatus* *A. schlegelii* *Hexagrammos otakii* *Agrammus agrammus*	-		-		7	*Trichiurus lepturus* *J. belangerii* *M. miiuy* *P. anomala* *P. argenteus* *L. polyacti* *C. lucidu* *Muraenesocis cinereus*
Cage culture area and nearby rock reefs	12	5	*S. marmoratus* *N. albiflora* *C. myriaster* *L. maculatus* *A. agrammus*	2	*N. albiflora* *Cynoglossus joyneri*	-		-	
Mussel culture area	19	4	*S. marmoratus* *J. belangerii* *L. polyacti* *N. albiflora*	-		-		-	

* The fish species names in this study are shown in Table 2.

## Data Availability

The data that support the findings of this study are available from the corresponding author upon reasonable request.

## References

[1] Dahlgren C., Marr J. (2004). Back reef systems: Important but overlooked components of tropical marine ecosystems. Bull. Mar. Sci..

[2] Wang K., Zhang S.Y., Wang Z.H., Zhao J., Jiang R.J. (2014). Dietary composition and feeding strategy of Agrammus agrammus off the Ma’an Archipelago special marine reserves. J. Shanghai Ocean. Univ..

[3] Liu H. (2014). Study on Bio-Resource Monitoring and Assessing Techniques and Aoolication in Offshore Island Area with Artificial Reefs.

[4] Huang H., Zhang Y.Y., Liu C.Y. (2020). Coral Reef Habitat and Resources Restoration in Tropical Island Marine Ranching. Top. Discuss..

[5] Ali A., Abdullah M.R., Safuan C.D.M., Afiq-Firdaus A.M., Bachok Z., Akhir M.F.M., Latif R., Muhamad A., Seng T.H., Roslee A. (2022). Side-Scan Sonar Coupled with Scuba Diving Observation for Enhanced Monitoring of Benthic Artificial Reefs along the Coast of Terengganu, Peninsular Malaysia. J. Mar. Sci. Eng..

[6] Wang Z.H., Gong F.X., Wu Z.L., Yuan X.B., Zhang S.Y. (2013). Efficiency of multi-mesh gillnets and multi-mesh trammel nets in collecting fish from rocky reef and sandy beach habitats. Chin. J. Ecol..

[7] Xu S.N., Wang Z.H., Liang J.L., Zhang S.Y. (2016). Use of different sampling tools for comparison of fish-aggregating effects along horizontal transect at two artificial reef sites in Shengs. J. Fish. China.

[8] Baldigo B.P., Sporn L.A., George S.D., Ball J.A. (2017). Efficacy of Environmental DNA to Detect and Quantify Brook Trout Populations in Headwater Streams of the Adirondack Mountains, New York. Trans. Am. Fish. Soc..

[9] Van Denderen P.D., Bolam S.G., Hiddink J.G., Jennings S., Kenny A., Rijnsdorp A.D., van Kooten T. (2015). Similar effects of bottom trawling and natural disturbance on composition and function of benthic communities across habitats. Mar. Ecol. Prog. Ser..

[10] Shan X.J., Li M., Wang W. (2018). Application of Environmental DNA Technology in Aquatic Ecosystem. Prog. Fish. Sci..

[11] Dejean T., Valentini A., Duparc A., Pellier-Cuit S., Pompanon F., Taberlet P., Miaud C. (2011). Persistence of Environmental DNA in Freshwater Ecosystems. PLoS ONE.

[12] Zhao J., Zhang S.Y., Zhou X.J., Chen Q.M. (2013). Comparative analysis of two sampling gillnets of rocky reef area in Gouqi Islands, Shengsi, Zhejiang. J. Fish. China.

[13] Tong W.J., Zhang S.Y. (2011). Preliminary study on distribution of fishery resources in LvHua-SanHeng sea area based on Kriging. J. Biol..

[14] Li M., Wei T.T., Shi B.Y., Hao X.Y., Xu X.Y., Sun H.Y. (2019). Biodiversity monitoring of freshwater benthic macroinvertebrates using environmental DNA. Biodivers. Sci..

[15] Doi H., Takahara T., Minamoto T., Matsuhashi S., Uchii K., Yamanaka H. (2015). Droplet Digital Polymerase Chain Reaction (PCR) Outperforms Real-Time PCR in the Detection of Environmental DNA from an Invasive Fish Species. Environ. Sci. Technol..

[16] Kelly R.P., Port J.A., Yamahara K.M., Crowder L.B. (2014). Using Environmental DNA to Census Marine Fishes in a Large Mesocosm. PLoS ONE.

[17] Takahara T., Ikebuchi T., Doi H., Minamoto T. (2019). Using environmental DNA to estimate the seasonal distribution and habitat preferences of a Japanese basket clam in Lake Shinji, Japan. Estuar. Coast. Shelf Sci..

[18] Thomsen P.F., Willerslev E. (2015). Environmental DNA—An emerging tool in conservation for monitoring past and present biodiversity. Biol. Conserv..

[19] West K.M., Stat M., Harvey E.S., Skepper C.L., DiBattista J.D., Richards Z.T., Travers M.J., Newman S.J., Bunce M. (2020). eDNA metabarcoding survey reveals fine-scale coral reef community variation across a remote, tropical island ecosystem. Mol. Ecol..

[20] Aglieri G., Baillie C., Mariani S., Cattano C., Calo A., Turco G., Spatafora D., Di Franco A., Di Lorenzo M., Guidetti P. (2021). Environmental DNA effectively captures functional diversity of coastal fish communities. Mol. Ecol..

[21] Wang Z.H. (2011). Fish Community Patterns in Meta-Habitat: A Case Study from Ma’an Archipelago.

[22] Wang Z.H., Zhao J., Wang K., Zhang S.Y. (2013). Fish community ecology in rocky reef habitat of Ma’ an Archipelago Ⅱ. Spatio-temporal patterns of community structure. Acta Ecol. Sin..

[23] Wang Z.H., Zhang S.Y., Chen Q.M., Xu Q., Wang K. (2012). Fish community ecology in rocky reef habitat of Ma’an Archipelago. I. Species composition and diversity. Biodivers. Sci..

[24] Zhao J., Zhang S.Y., Wang Z., Wang K. (2010). Analysis on community structure and diversity of fish and macroinvertebrate in Shengsi artificial reef area. J. Fish. Sci. China.

[25] Han X.D., Zhang S.Y., Wang Z.H., Wang K., Lin J., Deng M.Q., Wu X.C. (2019). Fish community structure and its relationship with environmental factors in the Ma’an Archipelago and its eastern waters. J. Fish. China.

[26] Boussarie G., Bakker J., Wangensteen O.S., Mariani S., Bonnin L., Juhel J.-B., Kiszka J.J., Kulbicki M., Manel S., Robbins W.D. (2018). Environmental DNA illuminates the dark diversity of sharks. Sci. Adv..

[27] Sigsgaard E.E., Nielsen I.B., Carl H., Krag M.A., Knudsen S.W., Xing Y., Holm-Hansen T.H., Moller P.R., Thomsen P.F. (2017). Seawater environmental DNA reflects seasonality of a coastal fish community. Mar. Biol..

[28] Stat M., Huggett M.J., Bernasconi R., DiBattista J.D., Berry T.E., Newman S.J., Harvey E.S., Bunce M. (2017). Ecosystem biomonitoring with eDNA: Metabarcoding across the tree of life in a tropical marine environment. Sci. Rep..

[29] Chen K., Fang C., Zhi-Gang W., Fan X., Dan Y., YongDe C. (2022). AeDNA: Aquatic Envionment DNA Database. Acta Hydrobiol. Sin..

[30] Edgar R.C. (2013). UPARSE: Highly accurate OTU sequences from microbial amplicon reads. Nat. Methods.

[31] Benson D.A., Clark K., Karsch-Mizrachi I., Lipman D.J., Ostell J., Sayers E.W. (2014). GenBank. Nucleic Acids Res..

[32] Lamy T., Pitz K.J., Chavez F.P., Yorke C.E., Miller R.J. (2021). Environmental DNA reveals the fine-grained and hierarchical spatial structure of kelp forest fish communities. Sci. Rep..

[33] Polanco Fernández A., Marques V., Fopp F., Juhel J.B., Borrero-Pérez G.H., Cheutin M.C., Dejean T., González Corredor J.D., Acosta-Chaparro A. (2021). Comparing environmental DNA metabarcoding and underwater visual census to monitor tropical reef fishes. Environ. DNA.

[34] Wang Z.H., Zhang S.Y., Wang K. (2010). Fish and macroinvertebrates community structure in artificial habitat around Sanheng Isle, Shengsi, China. Acta Ecol. Sin..

[35] Dixon P. (2003). VEGAN, a package of R functions for community ecology. J. Veg. Sci..

[36] Le Z.Y., Tang Y.W., Wang Z.Y. (2022). Reason analysis and resource protection suggestions on the frequent catch of wild large yellow croaker in Zhejiang Province sea area. Chin. Fish. Econ..

[37] Klymus K.E., Richter C.A., Chapman D.C., Paukert C. (2015). Quantification of eDNA shedding rates from invasive bighead carp Hypophthalmichthys nobilis and silver carp Hypophthalmichthys molitrix. Biol. Conserv..

[38] Ebisawa A., Kanashiro K., Ohta I., Uehara M., Nakamura H. (2016). Changes of group construction accompanying with growth and maturity in blue-barred parrotfish (*Scarus ghobban*), and influences of the fishing targeting the immature group to the stock. Reg. Stud. Mar. Sci..

[39] Wang Z.H., Liang J.L., Zhang S.Y. (2015). Comparison of pelagic and benthic fish assemblages in mussel farming habitat. J. Biol..

[40] Lin J., Deng M.X., Zhang S.Y., Yan Q. (2015). Seasonal variation of surface water temperature and its ecological impacts in a mussel aquaculture farm. J. Shanghai Ocean. Univ..

[41] Lin Y., Li J., Wang Z., Zhang S., Wang K., Li X. (2022). A Comparison of Fish Diversity in Rocky Reef Habitats by Multi-Mesh Gillnets and Environmental DNA Metabarcoding. Front. Ecol. Evol..

[42] Wang Z.H., Shen H., Lin J., Zhang S.Y., Zhong J.M., Chen Y.F., Liu Z.B. (2022). Spatial and temporal distribution of small yellow croaker (*Larimichthys polyactis*) in Eastern Ma’an Archipelago. J. Fish. China.

[43] Zamani N.P., Zuhdi M.F., Madduppa H. (2022). Environmental DNA biomonitoring reveals seasonal patterns in coral reef fish community structure. Environ. Biol. Fishes.

[44] Ling J.Z., Jiang Y.Z., Sun P., Yuan X.W., Zhang H., Tang B.J. (2021). Application and evaluation of environmental DNA technology in fish diversity research in Xiangshan Bay. J. Fish. Sci. China.

[45] Taberlet P., Coissac E., Pompanon F., Brochmann C., Willerslev E. (2012). Towards next-generation biodiversity assessment using DNA metabarcoding. Mol. Ecol..

[46] Zhong X.M., Tang J.H., Zhang H., Zhong F., Wu L., Gao Y.S. (2010). Temporal andspatial distributionof Miichthysmiiuy in Jiangsucoastal waters. Haiyang Xuebao.

[47] Luo B.Z. (1966). Seasonal growth the large yellow croaker, *Pseudosciaena crocea* (rich.), off chekiang. Oceanol. Limnol. Sin..

[48] Xu Z.L., Chen J.J. (2011). Analysis of migratory route *Larimichthys crocea* in the East China Sea and Yellow Sea. J. Fish. China.

[49] Wang X.Y., Lu G.Q., Zhao L.L., Du X.Q., Gao T.X. (2022). Assessment of fishery resources using environmental DNA: The large yellow croaker (*Larimichthys crocea*) in the East China Sea. Fish. Res..

[50] Lou B., Mao G.M., Shi H.L., Luo J., Jian X., Zheng D.M. (2008). Experiment on Artifi cial Domestication of Paralichthys olivaces (Temminck et Schlegel) Captured from Natural Waters along the Coast of Zhoushan. Fish. Inf. Strategy.

[51] Sato M., Inoue N., Nambu R., Furuichi N., Imaizumi T., Ushio M. (2021). Quantitative assessment of multiple fish species around artificial reefs combining environmental DNA metabarcoding and acoustic survey. Sci. Rep..

[52] Yu N.J., Yu C.G., Xu Y.J., Zheng J., Liu K., Zhang P.Y. (2021). Fish community structure and biodiversity in the offshore waters of Zhoushan Islands in spring and autumn. J. Fish. China.

[53] Xu Y., Ma L., Sun Y., Li X.Z., Wang H.F., Zhang H.M. (2019). Spatial variation of demersal fish diversity and distribution in the East China Sea: Impact of the bottom branches of the Kuroshio Current. J. Sea Res..

[54] Zhang S.Y., Liu S.R., Zhou X.J., Wang Z.H., Wang K. (2019). Ecological function of seaweed-formed habitat and discussion of its application to sea ranching. J. Fish. China.

[55] Zhang S.Y., Sun H.C. (2007). Research progress on seaweed bed ecosystem and its engineering. Chin. J. Appl. Ecol..

[56] Komatsu T., Mikami A., Sultana S., Ishida K., Hiraishi T., Tatsukawa K.-I. (2003). Hydro-acoustic methods as a practical tool for cartography of seagrass beds. Otsuchi Mar. Sci..

[57] Wang K., Zhang S.Y., Wang Z.H., Zhao J., Xu M., Lin J. (2012). Dietary composition and food competition of six main fish species in rocky reef habitat off Gouqi Island. Chin. J. Appl. Ecol..

[58] Diamant A., Tuvia A.B., Baranes A., Golani D. (1986). An analysis of rocky coastal eastern Mediterranean fish assemblages and a comparison with an adjacent small artificial reef. J. Exp. Mar. Biol. Ecol..

[59] Guo J., Wang T., Defang C., Yong L., Quan Q., Wang J. (2021). Feeding habits of *Acanthopagrus schlegeli* in the Daya Bay. J. Fish. Sci. China.

[60] Dong S., Xiuqing B., Chengqing Y. (2018). Analysis of Induced Corrosion by Fouling Organisms on Offshore Platform and Its Research Progress. Mater. Prot..

[61] Zhang S.Y., Xu M., Wang Z.H. (2010). Review of artificial reef and stock enhancement. Fish. Mod..

[62] Komyakova V., Chamberlain D., Swearer S.E. (2021). A multi-species assessment of artificial reefs as ecological traps. Ecol. Eng..

[63] Granneman J.E., Steele M.A. (2015). Effects of reef attributes on fish assemblage similarity between artificial and natural reefs. Ices J. Mar. Sci..

[64] Zhang R.L., Sun D.Y., Hou C.W., Zhao J.M. (2021). Characteristics of benthic fishery community at natural reefs and artificial reefs located in oddshore area. Oceanol. Limnol. Sin..

[65] Guo Y., Zhang S.Y., Cheng X.P., Lin J. (2020). Acoustic estimation of fisheries resources off Ma’an Archipelago. J. Fish. China.

